# Supplementary material for: Stable trapping of multiple proteins at physiological conditions using nanoscale chambers with macromolecular gates
Source: Nat Commun. 2023 Aug 23;14:5131. doi: 10.1038/s41467-023-40889-4 (PMC10447545; doi:10.1038/s41467-023-40889-4)
Supplement: Supplementary file 1 — Supplementary Information [file 41467_2023_40889_MOESM1_ESM.pdf]

Stable Trapping of Multiple Proteins at Physiological Conditions Using Nanoscale Chambers with Macromolecular Gates

Justas Svirelis,[1] *Zeynep Adali,*[1] *Gustav Emilsson,*[1] *Jesper Medin,*[1] *John Andersson,*[1] *Radhika Vattikunta,*[1] *Mats Hulander,*[1] *Julia Järlebark,*[1] *Krzysztof Kolman,*[1] *Oliver Olsson,*[1] *Yusuke Sakiyama,*[2] *Roderick Y. H. Lim*[2] *and Andreas Dahlin.*[1]

1 Department of Chemistry and Chemical Engineering, Chalmers University of Technology, 41296 Gothenburg, Sweden.

2 Biozentrum and the Swiss Nanoscience Institute, University of Basel, 4056 Basel, Switzerland.

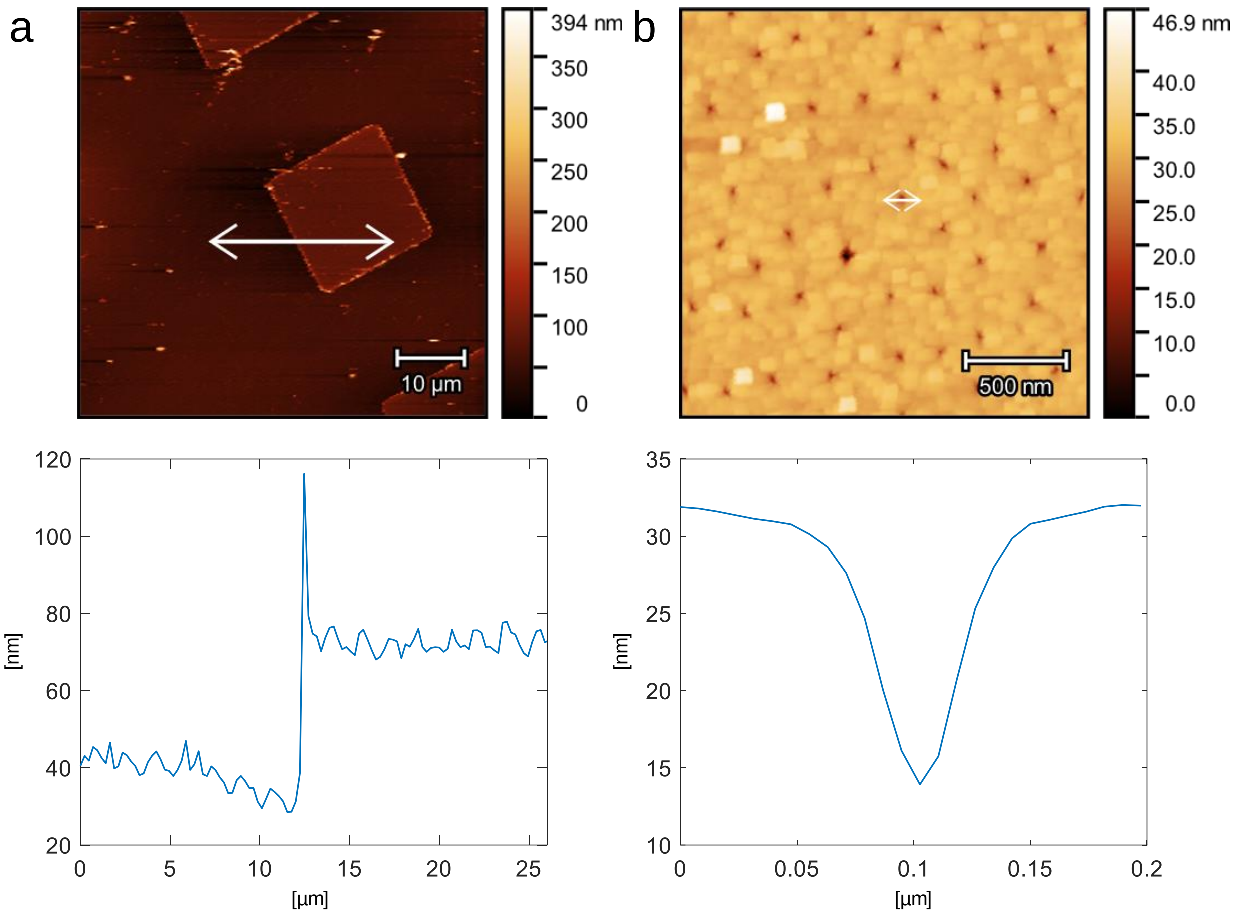

Supplementary Figure 1 Liquid-phase AFM imaging in contact mode to analyze polymer thickness at room temperature. (a) 20 nm thick gold "pixels". The line profile indicates a brush thickness of up to ~30 nm, while the expected exclusion height from SPR measurements was ~60 nm in this case. The lower height measured in AFM is attributed to the force setpoint, i.e. the imaging requires a certain compression of the brush. (b) Dense nanochamber array. The line profile shows that the tip can only penetrate slightly (~15 nm) into the apertures. Some variation in brush thickness over the planar gold regions is indicated, possibly suggesting non-uniformity. Note that these images were not obtained in the same manner as the high-speed AFM, which uses another instrument operating in tapping mode.

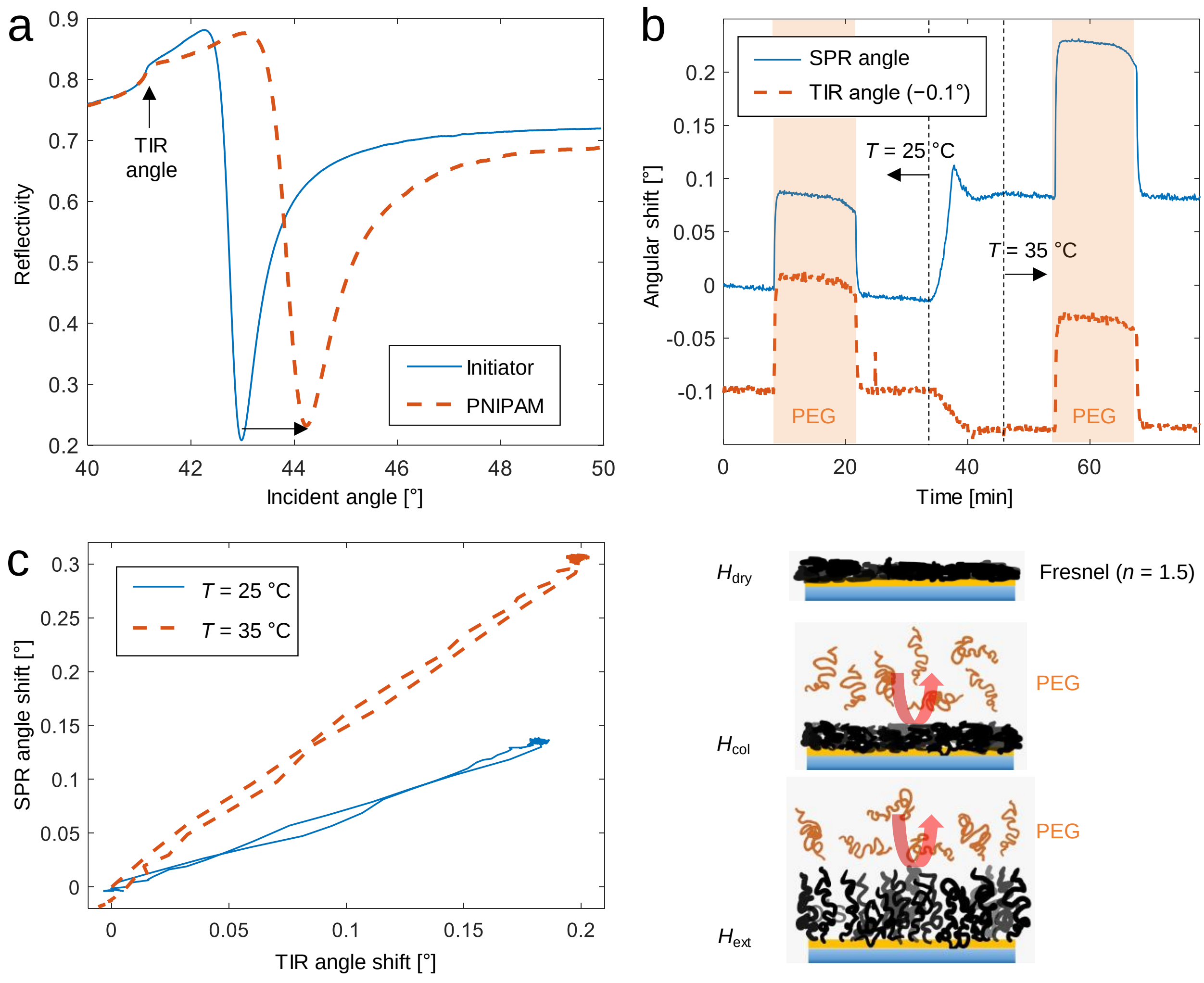

Supplementary Figure 2 PNIPAM brush heigh determination on planar gold surfaces by SPR. (a) Dry state spectra before and after polymerization. The thickness H_{dry} is determined by Fresnel models where the RI of PNIPAM is set to 1.5. (b) Example plots of SPR and TIR angles during injections of 35 kgmol^{-1} PEG at 10 gL^{-1}, before and after heating the system above the transition temperature. The TIR angle response is similar but the SPR angle response increases. The collapse of the brush is detected as a shift in the SPR angle, while heating of the water bulk causes the negative signal in the TIR angle. Fresnel models give the extended and collapsed brush heights (H_{ext} and H_{col}) based on the response from a non-interacting probe. (c) Example plot of SPR angle signal vs TIR angle signal during PEG injections to confirm a non-interacting probe.[1] Schematics show the principles for height determination. Abbreviations: SPR, surface plasmon resonance; TIR, total internal reflection, PEG, poly(ethylene glycol); PNIPAM poly(N-isopropylacrylamide).

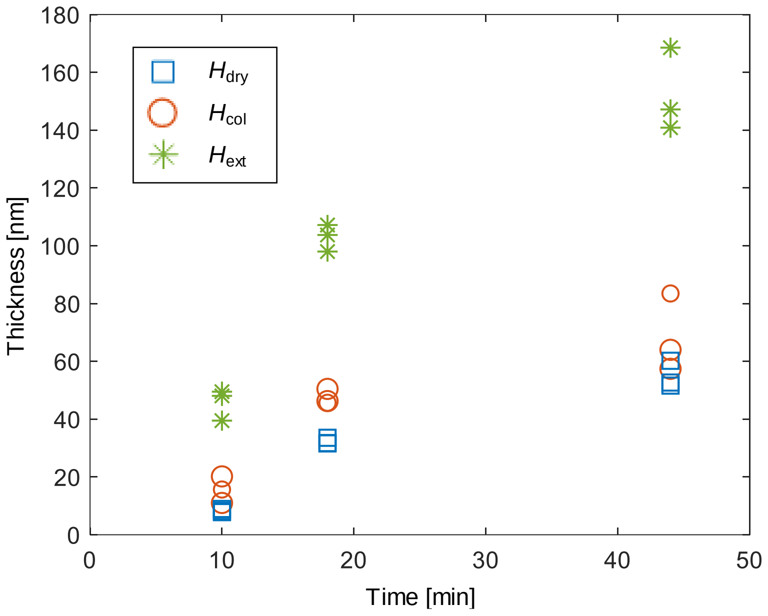

Supplementary Figure 3 Summary of the different brush heights measured by SPR for the most used polymerization recipe (60% molar faction water). Data from three different polymerization times are shown. Different surfaces and different batches were included to fully represent sample-to-sample variation (three values in each case). Note, however, that all chemicals were the same (supplier and batch) when generating this data set and variation may be larger if this is not the case. Termination is noticeable after 44 min. The initial "lag phase" indicated by the low values of the first data points at 10 min is attributed to the time it takes to fully introduce all reactants and mix them.

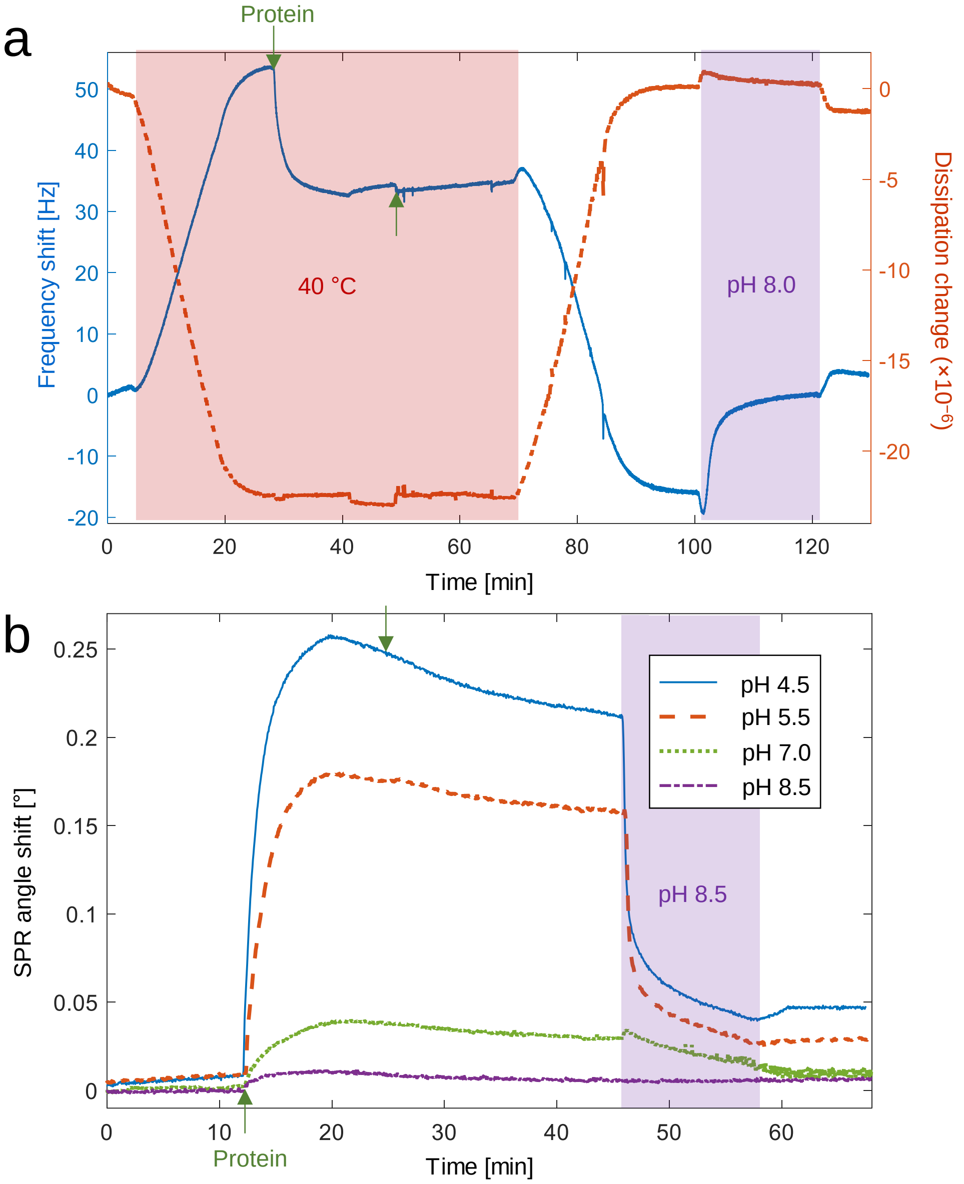

Supplementary Figure 4 Complementary SPR and QCM experiments on silica coated sensors. (a) BSA adsorption and desorption by pH increase on SiO_2 coated QCM sensor crystals. Arrows (on the frequency trace) indicate injection and rinsing. Note that the temperature is altered during the adsorption as in the nanoplasmonic measurements. Based on the Sauerbrey relation, which tends to overestimate the mass, the saturated coverage of BSA is around 350 ngcm^{-2}. (b) SPR data illustrating BSA adsorption tendencies at different pH (given in legend). Arrows (on the pH 4.5 trace) indicate injection and rinsing. The adsorbed amount is clearly lower at higher pH. For the lowest pH of 4.5, the binding kinetics are not monotonic, which is likely related to protein aggregation. (We never used such low pH in the other experiments.)

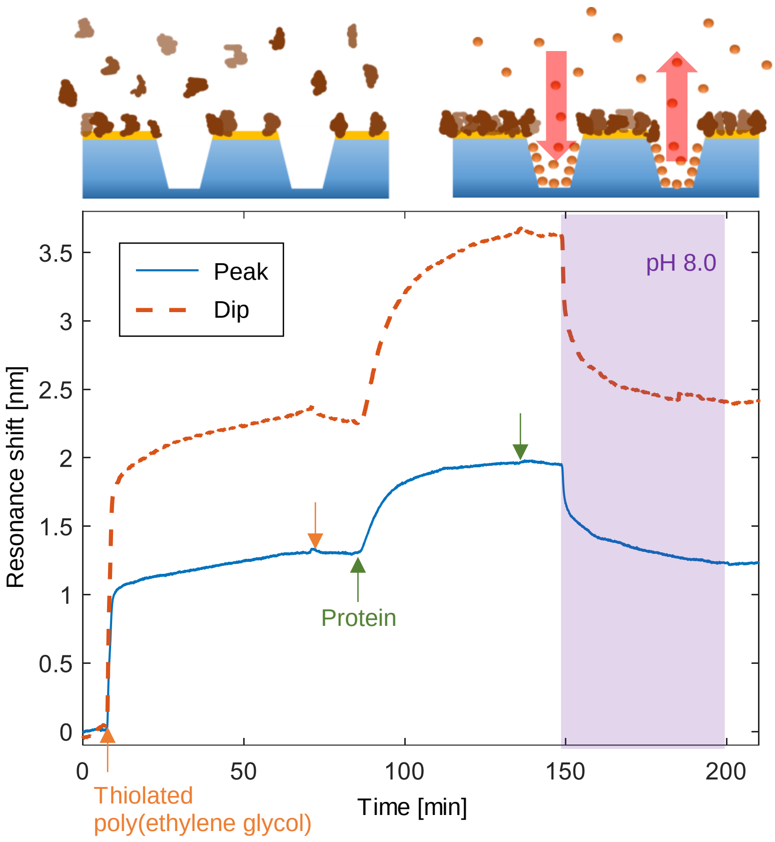

Supplementary Figure 5 Control experiment with plasmonic nanochambers to show adsorption and release of BSA, using a thin (~5 nm) PEG brush (2 kgmol^{-1} at 2 gL^{-1}) on gold which is known to be protein repelling.[2] The running buffer is PBS at pH 6.0. Arrows (along the peak trace) indicate injections and rinsing. Besides confirming full desorption of BSA upon raising the pH, one can also note that the binding kinetics are very similar to those measured when BSA adsorbs inside open PNIPAM nanochambers (Figure 3b in main text). This shows that the nanochambers are indeed "fully open" above the transition temperature, i.e. the proteins diffuse through unhindered. Furthermore, there cannot be a very high amount of protein adsorption onto the collapsed PNIPAM because then the signal magnitudes in peak and dip would be very different in this experiment when comparing with the PNIPAM-modified nanochambers (which is not the case).

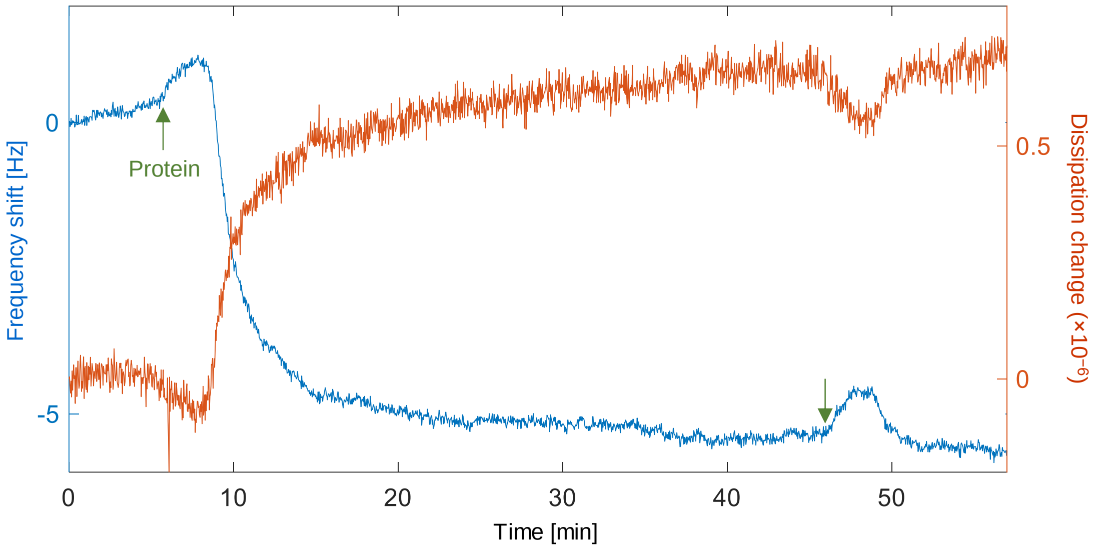

Supplementary Figure 6 QCM data showing response from BSA introduced to collapsed PNIPAM at pH 6.0. The temperature controller was set to 40 °C. Arrows (along the frequency trace) indicate injection and rinsing. The frequency and dissipation responses are much smaller than those on silica (Supplementary Figure 4a). The Sauerbrey relation, which is well-known to overestimate mass, gives a coverage <100 ngcm^{-2}. The proteins that do adsorb seem not to spontaneously desorb upon rinsing but should be removed upon cooling and brush hydration. This could not be clearly measured in the QCM because of the very much higher response from the brush collapse/extension[3] (hundreds of Hz). See instead Supplementary Figure 7 for confirmation of protein release from the gold regions.

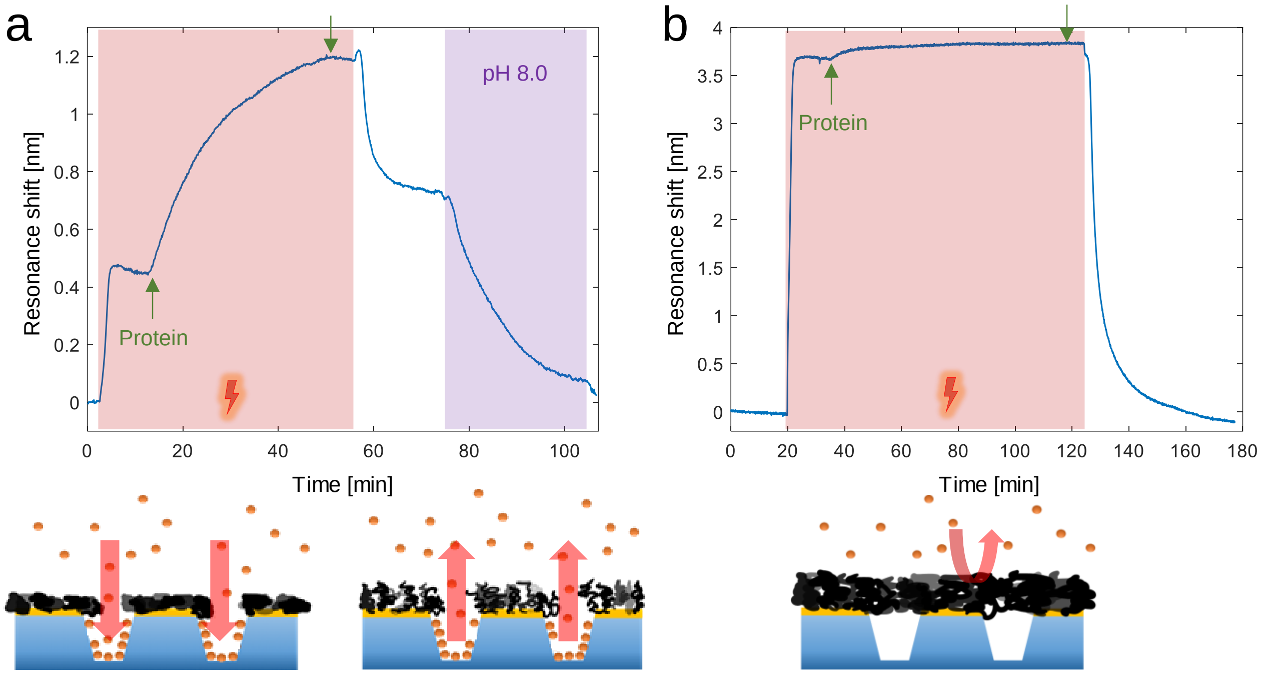

Supplementary Figure 7 Nanoplasmonic sensing (peak shift) of protein adsorption at pH 6.0 when the brush thickness is not suitable for trapping. (a) Too thin brush. Proteins leave the chambers after the pH increase step and the baseline is recovered, just like when there is a PEG coating on gold (Supplementary Figure 5). Note that the response from the brush collapse is much smaller (0.5 nm) compared to that in the main text (Figure 2b). (b) Too thick brush. The signal from protein adsorption is low (0.15 nm) and reversible with temperature, consistent with a small amount of secondary adsorption to the collapsed PNIPAM (Supplementary Figure 6). Note that the response from the brush collapse is much higher (3.7 nm) compared to that in the main text (Figure 2b).

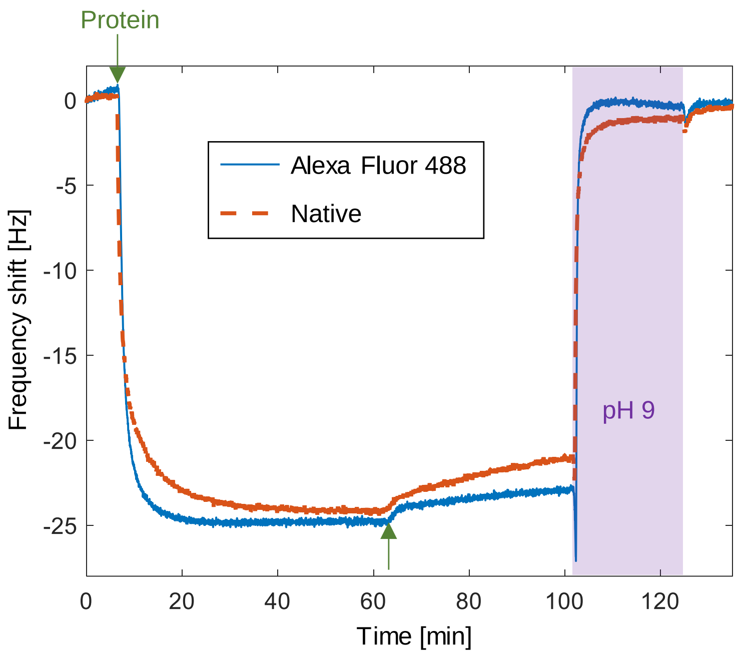

Supplementary Figure 8 Adsorption and desorption of native BSA vs labelled BSA. The running buffer has pH ≈ 6. No differences in adsorption and desorption behavior can be seen. The dissipation signals were negligible (~0.3 × 10^{-6}). Interestingly, we noticed some differences for BSA if it was instead labelled with fluorescein. This version seemed to adsorb in higher amounts, though it was also fully desorbed at high pH. Furthermore, we saw indications that for native BSA, the adsorption and desorption behavior was dependent on factors such as batch, product number or supplier. This could potentially be related to impurities, in particular other proteins. We emphasize that care must be taken to characterize the adsorption and desorption behavior of each protein (preferably using SPR or QCM) before trapping in nanochambers.

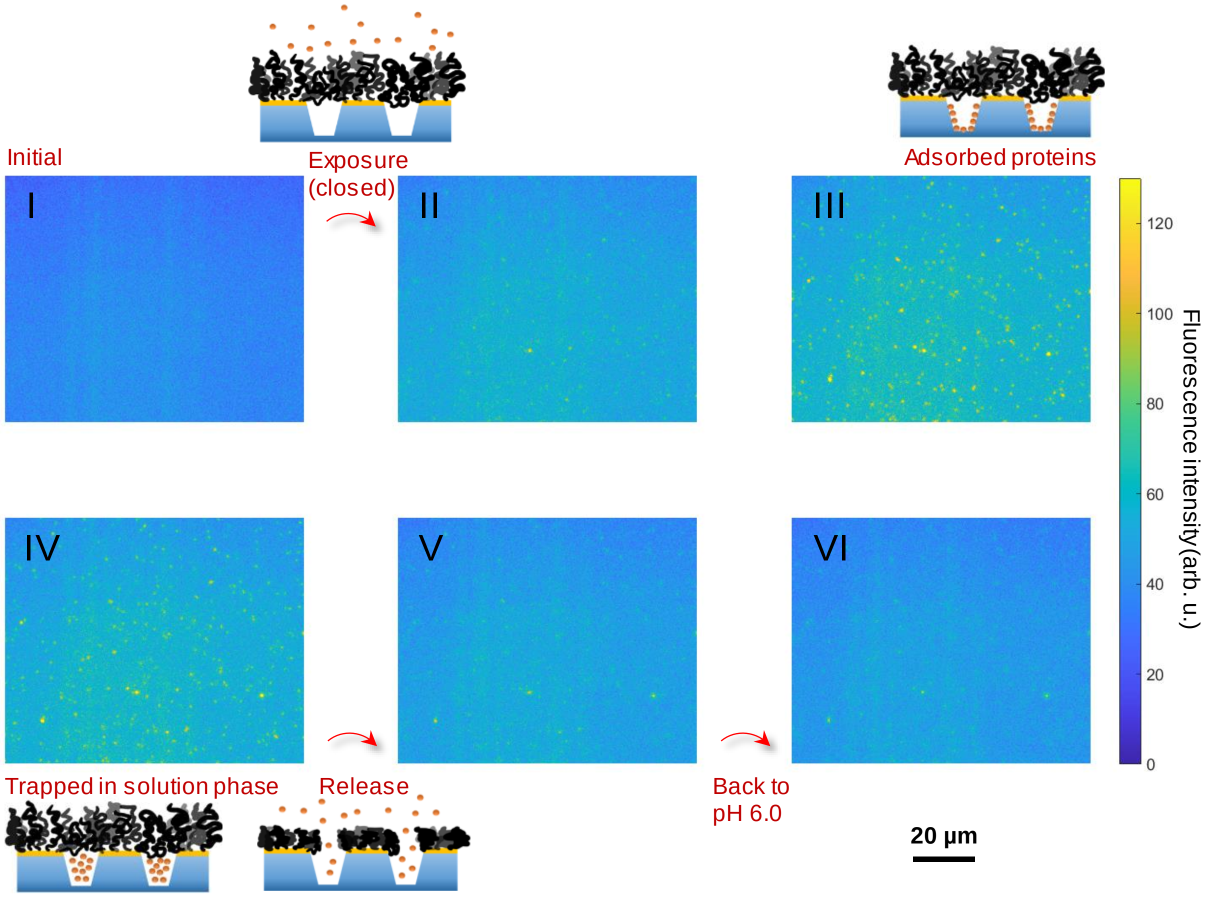

Supplementary Figure 9 Additional data showing fluorescence intensity maps from sparse nanochamber arrays during a whole trapping experiment. The color scale is the same for all images. The first image shows the intensity before any fluorescent protein has been introduced. The first step is a control where proteins are introduced at room temperature, when the gates should be closed. The last step is a control where only the pH is changed back to 6.0. Note that spots appear primarily in steps 3 and 4. All images were obtained without proteins in the bulk solution and at room temperature.

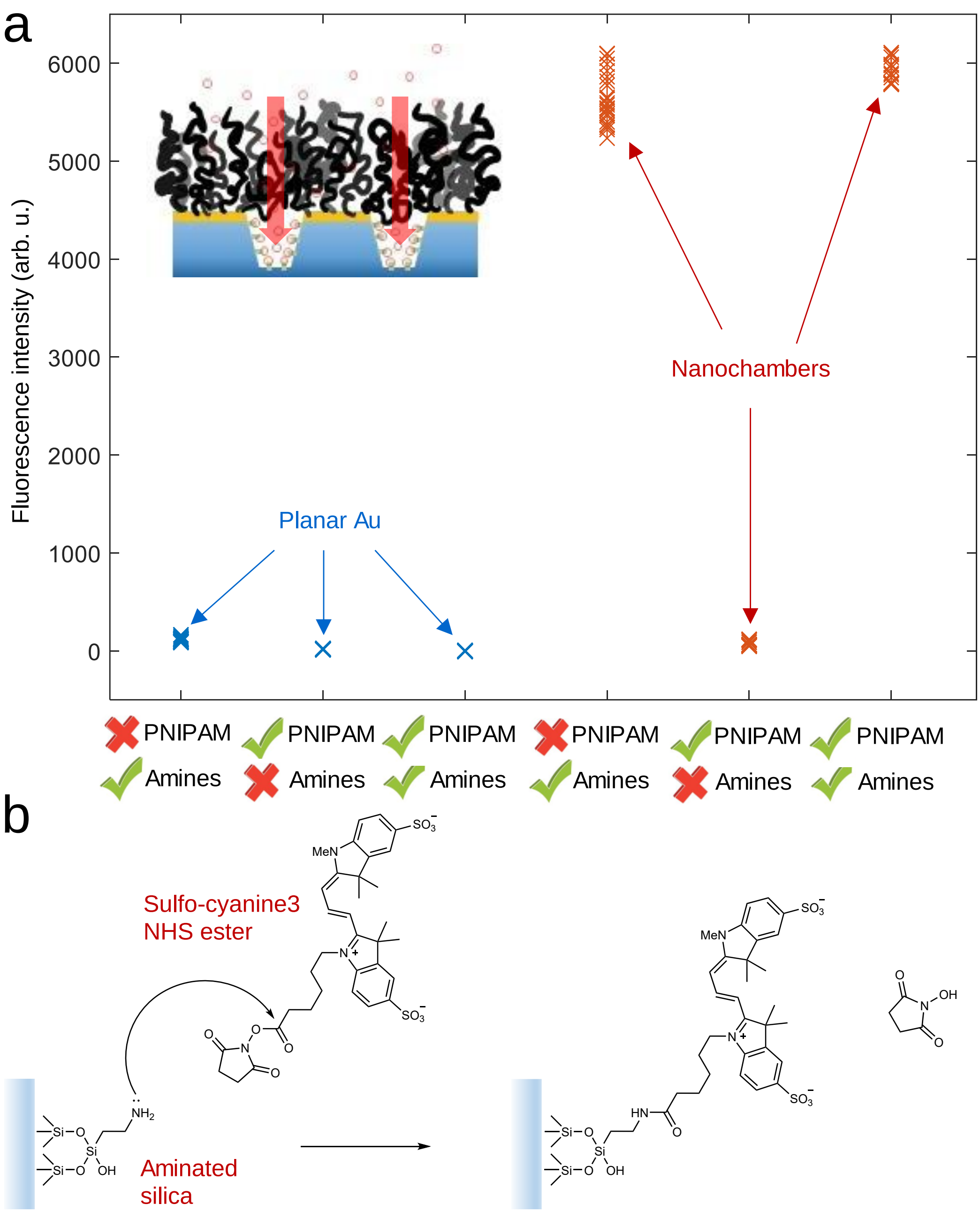

Supplementary Figure 10 Silanization and small molecule access to the "closed" nanochambers. (a) Fluorescence intensities (background subtracted) measured at RT from nanochambers and planar gold modified in different ways after exposure to 30 µM dye for 1 h and rinsing (24 data points for each of the six cases). A clear signal is only seen from nanochambers when they contain amine groups and it remains high for the case when a thick PNIPAM brush is present. Error bars represent variation from subsequent acquisitions (one per min). No significant fluorescence signal is observed when the interior is not aminated or from planar gold surfaces. The experiments were done with very thick PNIPAM brushes (>50 nm in dry state) to see any effects due to reduced transport of the dye. The results clearly show that the dye can diffuse through the brush barrier. (b) Reaction scheme for attachment of the dye.

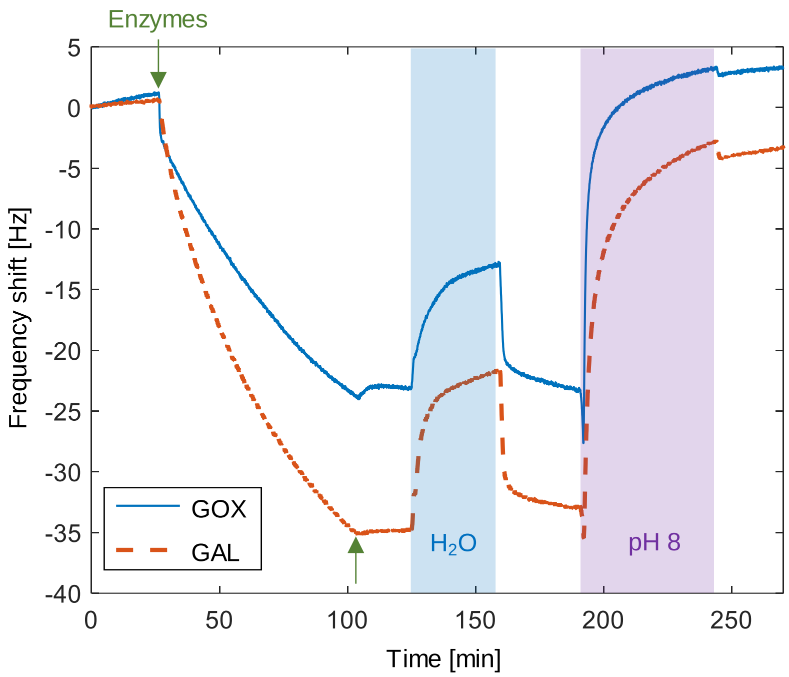

Supplementary Figure 11 QCM data verifying adsorption and desorption of β-galactosidase (GAL) and glucose oxidase (GOX) by changing pH. The running pH is 6. Arrows (along the GAL trace) indicate injection (50 μgmL^{-1}) and rinsing. The signals approach values that are slightly higher than for BSA (Supplementary Figure 8), which is because of the higher molecular weights (465 kg/mol for GAL and 160 kg/mol for GOX). The results are not surprising considering that the pI values for these enzymes are similar to BSA (4.6 for GAL and 4.2 for GOX). Note that injection of water mainly gives a signal from the changes in liquid bulk properties and not much protein desorption.

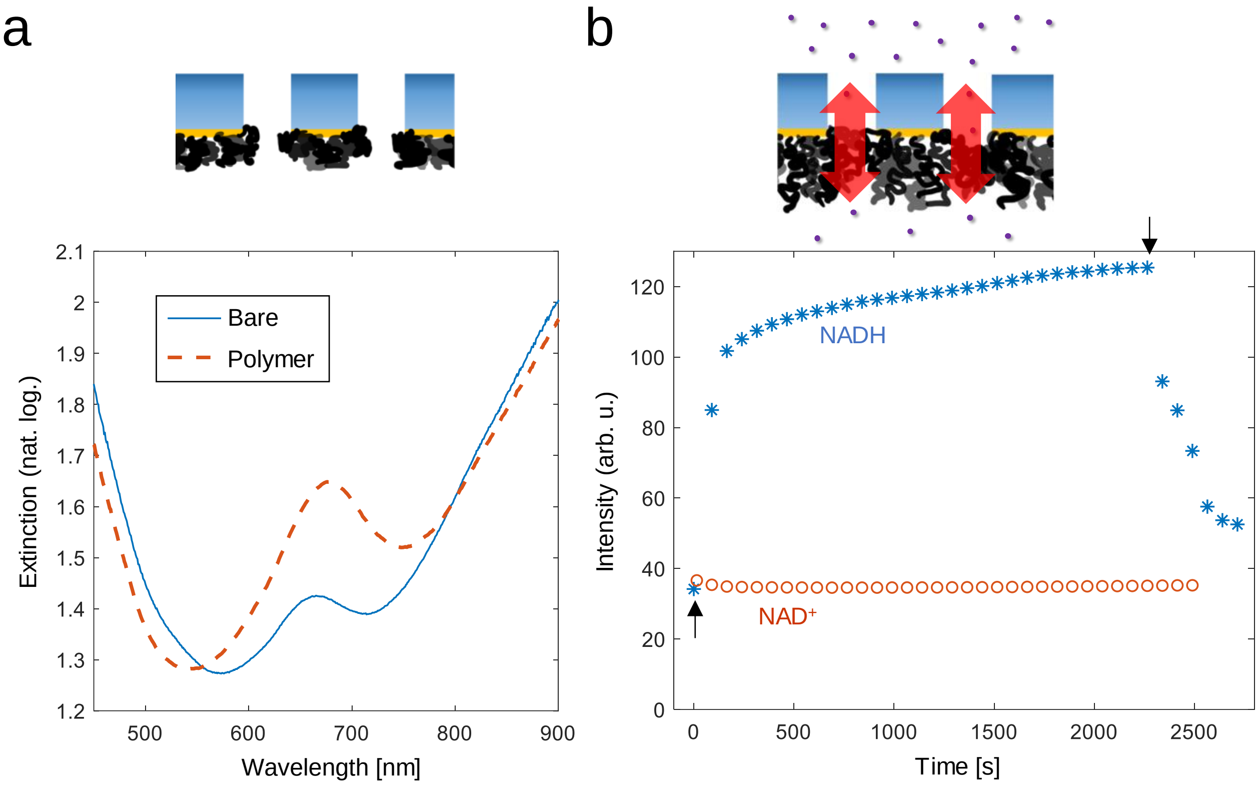

Supplementary Figure 12 Verifying transport of NADH through nanopores modified with polymer brushes (similar to the experiments in Figure 3c). (a) Extinction spectra of nanopore arrays to verify brush formation. (b) NADH was introduced on one side of the membrane and the fluorescence measured on the other side by excitation with a 375 nm diode. The fluorescence went down again when rinsing away the NADH on the feed side, showing that NADH diffuses back out of the reservoir. As a control, injecting NAD^+ did not generate any signal because it is not fluorescent.

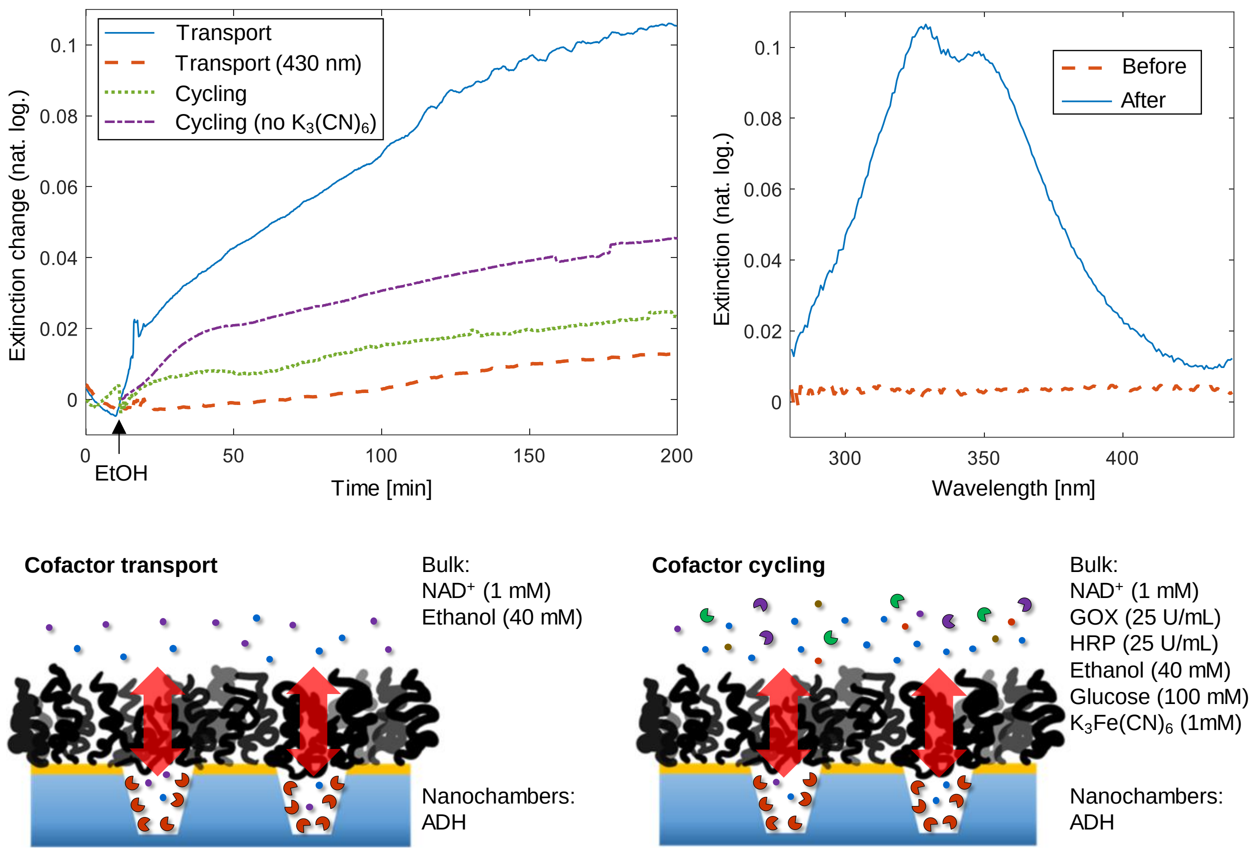

Supplementary Figure 13 Verifying cofactor transport and cycling with nanochambers. NAD^+ in solution is reduced by alcohol dehydrogenase (ADH) into NADH, which is detected by its absorbance at 340 nm. No significant increase was measured at 430 nm. This "cofactor transport" system proves that NAD^+ diffuses into the nanochambers and that NADH diffuses out. (If NADH would be stuck inside the nanochambers the reaction would stop and the absorbance change would be negligible.) Additionally, by including other enzymes in the liquid environment outside the chambers, a "cofactor cycling" system was created which quickly eliminates the NADH formed. Here GOX and glucose produces H_2O_2, while horseradish peroxidase (HRP) and ferricyanide are additional redox mediators since H_2O_2 alone does not oxidize NADH.[4] The effect from HRP without ferricyanide was noticeable, as expected,[5] but smaller. Conditions were chosen where ADH remains adsorbed using silica modified by silanization (Supplementary Figure 10b) and HEPES buffer at pH 7.5. In all cases, reactions are initiated by injecting the ethanol (which has no absorbance contribution) with all other components in place. The scheme is summarized in Supplementary Figure 14.

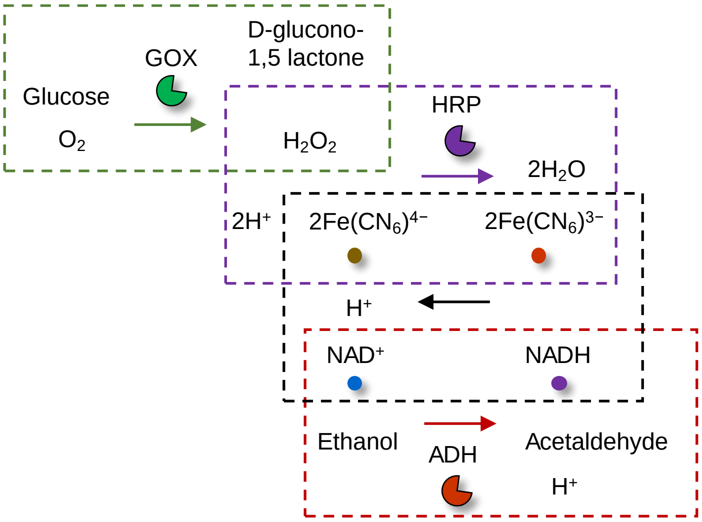

Ethanol + Glucose + O_2 → D-glucono-1,5 lactone + Acetaldehyde + $2H_2O$

Supplementary Figure 14 Proposed reaction scheme for NADH cycling with ALD, GOX and HRP. Each dashed box contains the species participating in one reaction. Note that three out of four reactions are enzymatic. Ferricyanide/ferrocyanide is included as an additional redox couple which reacts with both HRP[6] and NADH.[7] (This molecule is also cycled between two oxidation states.) The net reaction, which does not include NADH and ferricyanide (or the enzymes), is written at the bottom. Since all NADH produced is quickly converted back to NAD^+, there is no strong absorbance increase in this system (Supplementary Figure 13).

Supplementary Notes 1

Reason for the small negative signal upon pH increase using nanoplasmonic sensing.

For very thin brushes, the negative signal when the pH is increased is of course mainly due to molecules leaving the chambers (Supplementary Figure 7a). However, even when the brushes were closing the chambers at RT (no detectable signal from BSA), a small negative signal was always seen when desorbing the proteins from the nanochamber walls (Figure 4a in main text). Yet fluorescence data did not show any corresponding signal decrease at the pH increase step (Figure 4b), which suggests that this is an effect of the plasmonic readout method. We attribute these observations to the inhomogeneous near field distribution in the nanochambers.[8] When the proteins leave the chamber walls they are likely, on average, located in regions where the sensitivity to refractive index changes is lower. This view is consistent with the fact that the signal decrease was not instant but followed the desorption kinetics of BSA very well.

Supplementary Notes 2

Origin of the fluorescence signal from nanochambers (compared to planar gold) at RT.

In Figure 4b, a small but significant fluorescence intensity difference is observed when comparing nanochambers and planar gold after exposure to BSA at RT and pH 6. Since the amount of exposed gold is the same on both regions, it is tempting to attribute this signal to a fraction of nanochambers that are open at RT. However, the small intensity remains even after raising the pH when the chambers are open, where one would expect it to go away due to desorption. Although minor, the signal calls for an additional explanation, which we believe is a combination of the inability of the polymer brush to fully block protein adsorption to gold and differences in transmission through the two surface regions. Due to the high sensitivity of fluorescence detection compared to label-free plasmonic sensing, it would be surprising if there would be absolutely no fluorescence at all after exposing PNIPAM-modified gold to labelled BSA. Indeed, some proteins could be detected in between individual nanochambers on the sparse arrays (Figure 5a in main text), potentially associated with a few defects from the nanofabrication process (e.g. colloids that were not removed). Furthermore, the extinction of the dense nanochamber array is generally lower than for a gold film of the same thickness.[9] (This is not true at the resonance peak, but the fluorophore emits at shorter wavelengths.) Since the intensity is measured through the glass support, emitted light will pass through the gold and the plasmonic activity will therefore influence the intensity that reaches the detector from the nanochamber region. (The effect was clearly observed when there were fluorescent molecules in the liquid bulk, in which case a higher intensity went through the nanochamber region.) Thus, small amounts of protein adsorption on gold, potentially due to minor defects in the polymer coating, can explain the higher intensity measured through the nanowells.

Supplementary Notes 3

Reasons for (a small fraction of) malfunctioning chambers.

As mentioned in the main text, individual nanochambers not containing proteins after the pH increase step may be the result of the brush being (on average) either too high or too low (Figure 5a in main text). We could identify cases in the single nanochamber experiments where the brush was indeed much too thick or too thin by recording fluorescence from individual nanochambers during all the different steps in the trapping. Following Supplementary Figure 9, if the brush was generally too thick there were very few fluorescent spots correlating with nanowells in step III. Similarly, if the brush was generally too thin, many fluorescent spots correlating with nanowells appeared already in step II and disappeared in step IV. This is essentially the same findings as obtained from plasmonic dense arrays in Supplementary Figure 7. However, even when the polymerization time was around the optimum for creating the right brush thickness, we could still identify some nanochambers that did not capture proteins as intended. In these cases the results did not indicate a too thick or a too thin brush, but rather a mixture of both. This can be partly attributed to the size distribution of the nanochamber openings, but it is quite narrow with almost all the aperture diameters within ±10 nm.[9] Therefore, we also believe that non-uniformity of the PNIPAM brush is an important effect, i.e. that the thickness varies over the sample area even if the average value is indeed suitable for trapping. This is consistent with the liquid-phase AFM data (Supplementary Figure 1) which does not show a very smooth surface in between the nanochambers. In summary, although most nanochambers work as intended when the ATRP is performed carefully, future improvements should focus not only on fine control of the average brush thickness, but also its uniformity by varying factors such as reactant/catalyst concentrations and solvent composition.

Supplementary Notes 4

Protein denaturation and the importance of silanization.

As mentioned in the main text, BSA is known to not undergo any irreversible changes in structure due to adsorption to and desorption from silica through pH changes. This is likely the case for many proteins because, in general, protein denaturation occurs on hydrophobic surfaces where hydrophobic interactions with their interior makes them unfold. On silica, tunable electrostatic interactions can be used to cause adsorption that can be reversible with pH (or potentially salt) while the protein remains folded. However, it cannot be assumed that this approach will be non-invasive for all water-soluble proteins. Therefore, the possibility to use silanization of the nanochamber interior surface (Supplementary Figure 10) is very important. Proteins are even less likely to denature if adsorbed on an organic film. The broad range of silanization chemistries available, including recent improvements with respect to monolayer uniformity and click chemistry for further modification,[10] opens up for many possibilities to ensure that proteins are adsorbed and released in a gentle manner. It is even possible to release proteins adsorbed to the walls by conventional immobilization protocols, such as His-tags. The eluents used are normally small molecules (imidazole for His-tags) which can diffuse through the brush barrier. The salt content can be changed temporarily during such a desorption process. With such an approach, there is not even a need to change pH.

Supplementary References

1. Ferrand-Drake del Castillo, G., Emilsson, G. & Dahlin, A. Quantitative analysis of thickness and pH actuation of weak polyelectrolyte brushes. *The Journal of Physical Chemistry C* **122**, 27516-27527 (2018).
2. Malekian, B. et al. Fabrication and characterization of plasmonic nanopores with cavities in the solid support. *Sensors* **17**, 1444 (2017).
3. Emilsson, G. et al. Surface plasmon resonance methodology for monitoring polymerization kinetics and morphology changes of brushes - evaluated with poly(N-isopropylacrylamide). *Applied Surface Science* **396**, 384-392 (2017).
4. Lv, R. et al. Cascade cycling of nicotinamide cofactor in a dual enzyme microsystem. *Chemical Communications* **56**, 2723-2726 (2020).
5. Halliwell, B. & de Rycker, J. SUPEROXIDE AND PEROXIDASE-CATALYSED REACTIONS. OXIDATION OF DIHYDROXYFUMARATE, NADH AND DITHIOTHREITOL BY HORSERADISH PEROXIDASE*. *Photochemistry and Photobiology* **28**, 757-762 (1978).
6. Dunford, H.B. & Hasinoff, B.B. Kinetics of the oxidation of ferrocyanide by horseradish peroxidase compounds I and II. *Biochemistry* **9**, 4930-4939 (1970).
7. Komor, E., Thom, M. & Maretzki, A. The oxidation of extracellular NADH by sugarcane cells: Coupling to ferricyanide reduction, oxygen uptake and pH change. *Planta* **170**, 34-43 (1987).
8. Emilsson, G. et al. Nanoplasmonic sensor detects preferential binding of IRSp53 to negative membrane curvature. *Frontiers in Chemistry* **7**, 1 (2019).
9. Xiong, K., Emilsson, G. & Dahlin, A.B. Biosensing using plasmonic nanohole arrays with small, homogenous and tunable aperture diameters. *Analyst* **141**, 3803-3810 (2016).
10. Andersson, J. et al. Polymer brushes on silica nanostructures prepared by aminopropylsilatrane click chemistry: superior antifouling and biofunctionality. *ACS Applied Materials & Interfaces* **15**, 10228-10239 (2023).
