# Supplementary material for: Stable trapping of multiple proteins at physiological conditions using nanoscale chambers with macromolecular gates
Source: Nat Commun. 2023 Aug 23;14:5131. doi: 10.1038/s41467-023-40889-4 (PMC10447545; doi:10.1038/s41467-023-40889-4)
Supplement: Supplementary file 3 — Description of Additional Supplementary Information [file 41467_2023_40889_MOESM3_ESM.pdf]

Description of Additional Supplementary Information File

Title: Supplementary Movie 1

Description: High-speed AFM data showing a closed macromolecular gate (room temperature).

Title: Supplementary Movie 2

Description: High-speed AFM data showing an open macromolecular gate (heated).
