# Supplementary figures and images for: Stable trapping of multiple proteins at physiological conditions using nanoscale chambers with macromolecular gates
Source: Nat Commun. 2023 Aug 23;14:5131. doi: 10.1038/s41467-023-40889-4 (PMC10447545; doi:10.1038/s41467-023-40889-4)

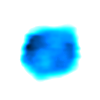

Supplement: Supplementary file 4 — Supplementary Movie 1 [file 41467_2023_40889_MOESM4_ESM.gif]

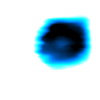

Supplement: Supplementary file 5 — Supplementary Movie 2 [file 41467_2023_40889_MOESM5_ESM.gif]
